# A systematic literature review of AI-based digital decision support systems for post-traumatic stress disorder

**DOI:** 10.3389/fpsyt.2022.923613

**Published:** 2022-08-09

**Authors:** Markus Bertl, Janek Metsallik, Peeter Ross

**Affiliations:** Department of Health Technologies, School of Information Technologies, Tallinn University of Technology, Tallinn, Estonia

**Keywords:** decision support systems (DSS), post-traumatic stress disorder (PTSD), artificial intelligence (AI), machine learning (ML), systematic literature review (SLR), clinical decision support (CDS), psychiatry, mental health

## Abstract

**Objective:**

Over the last decade, an increase in research on medical decision support systems has been observed. However, compared to other disciplines, decision support systems in mental health are still in the minority, especially for rare diseases like post-traumatic stress disorder (PTSD). We aim to provide a comprehensive analysis of state-of-the-art digital decision support systems (DDSSs) for PTSD.

**Methods:**

Based on our systematic literature review of DDSSs for PTSD, we created an analytical framework using thematic analysis for feature extraction and quantitative analysis for the literature. Based on this framework, we extracted information around the medical domain of DDSSs, the data used, the technology used for data collection, user interaction, decision-making, user groups, validation, decision type and maturity level. Extracting data for all of these framework dimensions ensures consistency in our analysis and gives a holistic overview of DDSSs.

**Results:**

Research on DDSSs for PTSD is rare and primarily deals with the algorithmic part of DDSSs (*n* = 17). Only one DDSS was found to be a usable product. From a data perspective, mostly checklists or questionnaires were used (*n* = 9). While the median sample size of 151 was rather low, the average accuracy was 82%. Validation, excluding algorithmic accuracy (like user acceptance), was mostly neglected, as was an analysis concerning possible user groups.

**Conclusion:**

Based on a systematic literature review, we developed a framework covering all parts (medical domain, data used, technology used for data collection, user interaction, decision-making, user groups, validation, decision type and maturity level) of DDSSs. Our framework was then used to analyze DDSSs for post-traumatic stress disorder. We found that DDSSs are not ready-to-use products but are mostly algorithms based on secondary datasets. This shows that there is still a gap between technical possibilities and real-world clinical work.

## Introduction

According to Sauter, Digital Decision Support Systems (DDSSs) are computer-based systems that bring together information from various sources, assist in the organization and analysis of information and facilitate the evaluation of assumptions underlying the use of specific models (1). The concept of decision support systems originated in the 1960s (2) when researchers began to study computerized methods to assist in decision-making (3–5). Since then, the idea has extended throughout a broad spectrum of domains, one of which is healthcare. This work focuses on decision support systems in mental health, more precisely on decision support systems for PTSD. The American Psychiatric Association defines PTSD as “a psychiatric disorder that can occur in people who have experienced or witnessed a traumatic event such as a natural disaster, a serious accident, a terrorist act, war/combat, rape or other violent personal assault” (6). People with PTSD experience recurrent thoughts about their traumatic experience that influence their daily life. The lifetime prevalence of PTSD is around 12.5% (7). However, people suffering from PTSD are often undiagnosed or misdiagnosed, resulting in incorrect, incomplete or missing treatment (8). To investigate whether DDSSs could be a solution to this problem, we aim to review available decision support systems for PTSD and map their technological approaches in order to understand possible research gaps and obstacles in introducing decision support systems to clinical processes. Since no available reference architecture for decision support systems is applicable to our research, we contribute by introducing a novel framework for decision support systems that can be used to analyze existing systems. Ultimately, this also accelerates the development of new systems by highlighting essential dimensions.

Designers of earlier DDSSs have applied multiple alternative approaches for converting real-world data into something that stimulates better decisions. Information-management-based DDSSs try to organize data into usable presentations; modeling-(or data-analytics)-based DDSSs attempt to apply statistical (learning) methods for finding patterns or calculating indicators; and knowledge-management-based systems apply externally prepared algorithms (expert rules) to find matching data or derive new facts (9). While AI has been an essential element of DDSSs throughout its history, only recently has a new generation of decision support been facilitated by the availability of powerful computing tools to properly manage big data and to analyze and generate new knowledge. The evaluation of AI’s earlier implementations was limited to the design and development phase; machine learning-based algorithms often do not generalize beyond the training data set (10). However, studies have still shown the benefits of machine learning algorithms in DDSSs (11–13). Current studies that test the application of healthcare AI algorithms often omit details of DDSS tools that apply AI models. A well-designed DDSS is likely to enable the real-world application of AI technology (14).

This review aims to contribute by introducing a framework for the features of DDSS implementation in mental health. We aim to identify the prevalent features of the current state of research on DDSS. Often, the development of information systems involves the continuous introduction of new features and quality improvements. We hypothesized that each available article presents only a selection of features, a selection which is dependent on the maturity of the DDSS. Maturity models are increasingly used as a means of benchmarking or self-assessment of development (15). In healthcare informatics, many maturity models are available [e.g., Hospital Information System Maturity Model (16)], but none of these models strictly provides an informed approach for the assessment of research on decision support systems (17). The available maturity models instead tend to look at the level of organizational adoption of specific technologies (e.g., how much an organization values data analytics technology) and provide little support for deciding on the readiness of DDSS tools in their early phases of development. As AI is often an essential element of a DDSS, we also explored AI maturity models. AI maturity models mostly look into the level of AI adoption in an organization rather than the maturity of the AI technology itself (18–20).

A DDSS is not a single technology but rather a set of integrated technologies (21–25). Sauser et al. (26) suggested a measure of System Readiness Level (SRL), which expresses the level of maturity of a system consisting of a set of integrated technologies (26). Exploring AI technology readiness or maturity, we encountered suggestions to look separately into the AI system’s capacities of integrating existing data sources (machine-machine intelligence), interacting with human users (human-computer intelligence) and applying intelligent reasoning (core cognitive intelligence) (27).

## Methods

To have a transparent and objective approach for this literature review, we decided to apply the five stages suggested by Kitchenham’s “Guidelines for performing Systematic Literature Reviews in Software Engineering” (28):

(1) Search Strategy
(2) Study Selection
(3) Study Quality Assessment
(4) Data Extraction
(5) Data Synthesis

### Research questions

Since our aim is to understand current research on decision support systems for PTSD, this paper is based on two research questions. First, we look for state-of-the-art decision support systems for post-traumatic stress disorder (RQ1). Second, we investigate the component elements of current decision support systems for PTSD (RQ2).

### Search strategy

We built a search string based on the research questions identified and applied it to the Scopus abstract and citation database. Scopus was chosen as the primary source because it is the largest abstract and citation database of research literature with 100% MEDLINE coverage (29). The initial search string consisted of the disease to investigate – post-traumatic stress disorder – its abbreviation PTSD as well as the term “decision support.” To find papers that covered the prediction and classification of PTSD, we also added Artificial Intelligence. In Scopus, we applied the search string to the title, abstract and tags of the research papers. We restricted our search to only include journal articles or conference proceedings in English. We also conducted a manual search using Google Scholar and the web to find additional research; however, this did not bring up any new articles not already covered by our database search and our reference screening process. We formed our search criteria as (“decision support” OR “Artificial Intelligence”) AND [PTSD OR (post AND traumatic AND stress AND disorder)].

**FIGURE 1:**
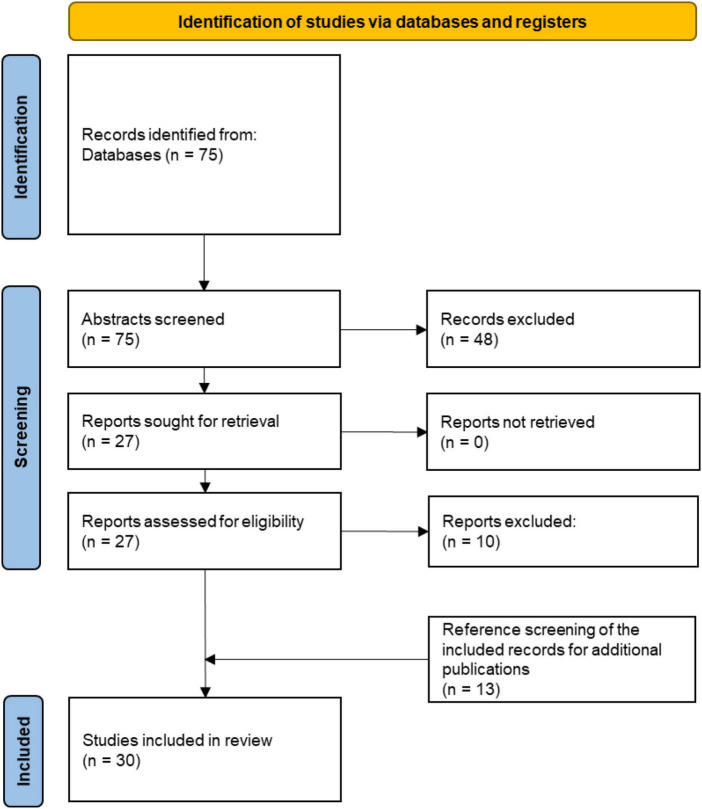
Search strategy.

We conducted the search in Scopus on 3 March 2021. It resulted in 75 papers; reference screening of the included literature brought up an additional 13 papers. Our search process is visualized in Figure 1.

### Study selection

The titles and abstracts of the queried articles were analyzed to identify relevant articles from the results of the search string queries. Articles fitting the research questions and meeting the inclusion criteria (see section “inclusion criteria”) as well as the quality criteria (see section “study quality assessment”) were included. Since the goal of this research is to give an overview of the state of the art, we did not put any constraints on study types and designs. To reduce bias in the study selection process, the task was done by two researchers independently. The two result sets were then merged and deviations were discussed among the authors. This resulted in a total set of 17 research papers.

We then repeated this process step to extract relevant studies from the reference lists of the selected articles. This resulted in 13 new research papers.

#### Inclusion criteria

Table 1 presents the inclusion criteria applied to the articles in our review (Inclusion criteria).

**TABLE 1:** Inclusion criteria.

#	Inclusion criteria
IC1	Does the study deal with decision support systems (e.g., systems that help diagnose, screen, predict or treat)
IC2	Does this study apply a computerized algorithm?
IC3	Does this article deal with PTSD?
IC4	Is the article related to at least one of our research questions?

#### Study quality assessment

Table 2 presents the inclusion criteria applied to the articles in our review (Quality criteria).

**TABLE 2:** Quality criteria.

#	Quality criteria
QC1	Is the research a journal article or conference proceeding?
QC2	Is the research peer-reviewed?
QC3	Does the study have a well-defined structure?
QC4	Does the study bring evidence for the proposed approach (either by citing relevant literature or validating the results)?
QC5	Does the study have ethics approval (if required by the study design)?

### Data extraction and synthesis

**FIGURE 2:**
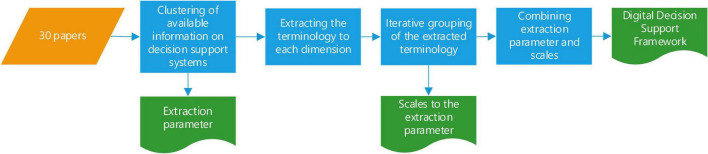
Extraction process.

Data extraction and synthesis were based on an inductive approach. We applied thematic analysis (30) to answer our research questions. First, clear, scoped questions for data extraction were formed. Two researchers read through all the articles and iteratively clustered all of the information available on decision support systems into the extraction parameters. These extraction parameters describe how decision support systems work. This process is shown in Figure 2.

The answers extracted from the EQs (see Table 3) were then combined upon the agreement of the authors to create a feature matrix. The extracted features were then further clustered to create a common terminology that allows further analysis and the possibility to compare results. In the end, we combined the developed extraction questions and the clustered scales of each question into a novel framework for decision support systems in mental health.

**TABLE 3:** Extraction questions (EQ).

#	Extraction parameters
EQ1	On the basis of which input data do existing decision support systems in mental health operate?
EQ1.2	What was the data sample size?
EQ2	What is the implementation technology of the DDSS?
EQ2.1	Decision technology
EQ2.2	User Interaction/Interface/Application
EQ2.3	Data collection technology
EQ3	What feature was validated?
EQ4	Which user groups are involved in the use of DDSS in mental health?
EQ5	What diseases are currently targeted by DDSS in mental health?
EQ6	What decisions are supported by the system?
EQ7	What maturity level does the DDSS have?

## Results

The selected 30 research articles (31–60) were published between 2001 and 2019. Three articles were published in journals about medical informatics, 10 in computer science journals or proceedings and 17 in medical journals. The following table shows how often each extraction parameter was present and indicates the terminology used in the selected studies. The terminology shown in Table 4 was developed by manual, iterative clustering of the extracted features until the authors were satisfied with the granularity.

**TABLE 4:** Terminology extraction.

EQ	Number of mentions	Terminology (frequency)
1 – Data	30	Jerusalem Trauma Outreach and Prevention Study (3); checklist (5); questionnaire (4); speech data (4); text data (3); electronic health records (3); sensor data (6); reactions in VR (2)
1.1 – Sample size	28	Not applicable (quantitative features)
2.1 – Decision technology	27	Machine learning algorithm; feed forward neural network; support vector machines, random forest; decision tree; sequential minimal optimization (SMO); Naïve Bayes; logistic regression; text mining; (LIWC); rule based
2.2 – Interaction technology	24	Questions (3); temperature control (1); aromatherapy (1); auditory therapy (1); virtual human (2); online survey (1); role-play-game (1); virtual reality (2)
2.3 – Data collection technology	22	Mobile app (4); web portal (3); skin conductance sensor (1); heart rate (1); accelerometer (1); IoT devices (1); microphone (1); webcam (1); Kinect (1); VR headset (1)
3 – Validation	29	Accuracy (23); user acceptance (3); efficacy (2)
4 – User groups	12	Patients (10); supporters (1); clinicians (6)
5 – Disease	30	PTSD (30); depression (4); anxiety (1); PTSD comorbidities (1); paranoia (1)
6 – Decisions	29	Prediction (11); assessment (1); diagnosis (4); screening (6); monitoring (5); treatment (6)
7 – Maturity level	30	Not applicable (quantitative features)

### A framework for digital decision support systems

**FIGURE 3:**
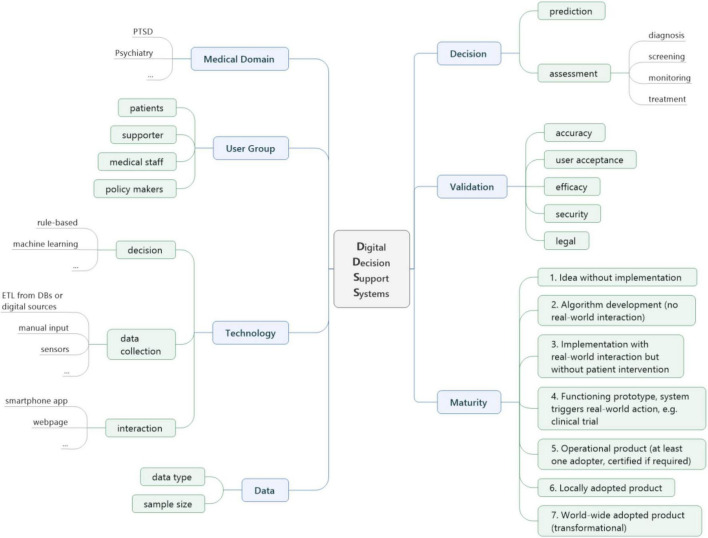
Framework for DDSS.

Based on our aim to find all relevant features of decision support systems in the PTSD area and our systematic literature review results, we propose a multidimensional framework that covers the different areas of DDSS. Each dimension represents one of our extraction parameters. Figure 3 illustrates our framework with the different dimensions of DDSSs. Based on the extracted data, we clustered the terminology to develop scales for dimensions in order to make results better analyzable.

**Input Data:** The input data dimension defines the information needed by a decision support system in order to function. Possible data could be structured like socio-demographic information or coded data [for example, with the International Statistical Classification of Diseases and Related Health Problems (ICD) (61) or the Diagnostic and Statistical Manual of Mental Disorders (DSM) (62)] as well as semi-structured information like patient records or unstructured information like free text or medical images. A combination of different structured, semi-structured and/or unstructured data is also possible.

**Technology:** The technology dimension describes how the decision support system is implemented. This involves three sub-dimensions:

**Decision technology:** The decision technology explains the intelligence of the cognition of the system. This is the algorithm that powers the decision-making. Examples are different machine learning algorithms such as support vector machines or other statistical methods as well as rule-based approaches.

**Interaction technology:** This sub-dimension describes the technology needed to interact with other systems or user groups in the clinical process. Interaction technology can be API-based interfaces to systems, graphical user interfaces (websites, mobile apps) or sensory input like conversational interfaces (chatbots).

**Data collection technology:** The data collection technology sub-dimension defines how the data described in the input data dimension are collected. Examples are instance sensors, questionnaires or chatbots.

**Validation:** Validation describes how the success of decision support systems is measured.

**Accuracy:** The decision support system is evaluated by how many right or wrong decisions it makes. Examples are accuracy, recall (sensitivity), precision, specificity, area under the curve (AUC) values and F1 scores (harmonic mean of recall and precision).

**User acceptance:** End-users are involved in the evaluation of the DDSS.

**Efficacy:** The impact of the decision support system is evaluated based on potential benefits.

**Security:** The DDSS is evaluated against security regulations.

**Legal:** The legal compliance of the DDSS is evaluated.

**User group:** This dimension captures the different user groups interacting with the decision support system in the clinical process.

**Medical domain:** The medical domain dimension describes the disease for which the decision support system can be applied.

**Decision:** The following scale defines the decisions a digital decision support system can support:

**Prediction:** The system outputs a risk score based on the likelihood that someone gets a disease.

**Assessment:** The patient is already sick (knowingly or unknowingly).

**Diagnosis:** Testing individuals with symptoms and/or suspicion of illness

**Screening:** Testing for individuals without specific symptoms

**Monitoring:** Decision support that evaluates symptom severity or treatment progress

**Treatment:** Recommendation or intervention concerning care or therapy

**Maturity:** As none of the existing maturity models fits our research, we designed a DDSS maturity model based on the SLR scale (26), but with adaptions specific to healthcare. It introduces additional gradation for noticing the moment where human interaction is added to the core AI algorithm. Our maturity levels describe on a scale from one to seven how advanced the DDSS is. Not all of the abovementioned dimensions are necessarily present in each of the maturity levels. As the maturity level gets higher, more dimensions are described.

1. Idea without implementation
2. Implementation without real-world interaction (algorithm development)
3. Implementation with real-world interaction but without patient intervention
4. Fully functioning prototype, system triggers real-world action, e.g., clinical trial
5. Operational product (at least one adopter, certified if required)
6. Locally adopted product
7. World-wide adopted product (transformational).

### Data synthesis input data (EQ1)

**FIGURE 4:**
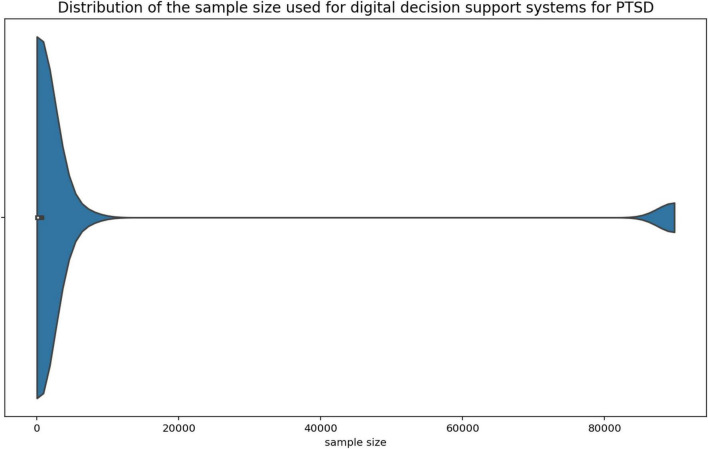
Sample size distribution.

**FIGURE 5:**
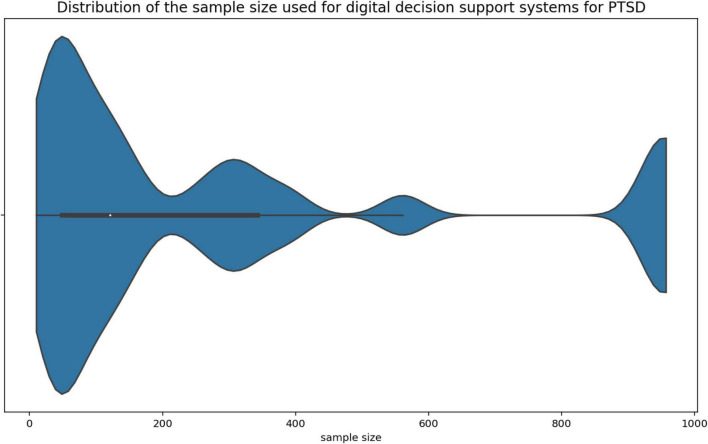
Sample size distribution excluding outliers.

The data used by digital decision support systems in the context of PTSD is diverse. Voice data (35, 45, 46, 55), text data (38, 48, 50), checklists and questionnaires (32, 33, 37, 41–43, 52, 53, 59), bio signals (32, 33, 36, 44, 45, 51, 57) and electronic medical records (34, 47, 56) as well as secondary data from other clinical studies (31, 40, 49, 54) are used. One article used the choices made by a virtual avatar in a role-playing game as input data (39). Of the 30 publications included in this review, 28 mentioned the sample size of the data they used to develop and test their decision support system. The minimum sample size was 10, and the maximum was 89,840 with a median (IQR) *m* = 151.5 (54.25 to 656.25). The violin plots (Figures 4, 5) below show the distribution of the sample size. The top three outliers (89,840; 89,840; 5,972) were neglected in Figure 5 for better visibility.

**FIGURE 6:**
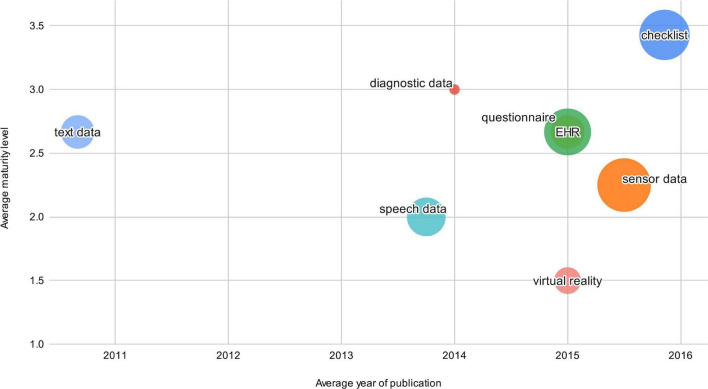
Data dimension concepts.

Figure 6 shows the data dimension of the studies in our review and indicates how the data used correlate with the average maturity levels of the DDSS. It visualizes the frequency and maturity of DDSSs based on the different data sources.

### Data synthesis implementation (EQ2)

**FIGURE 7:**
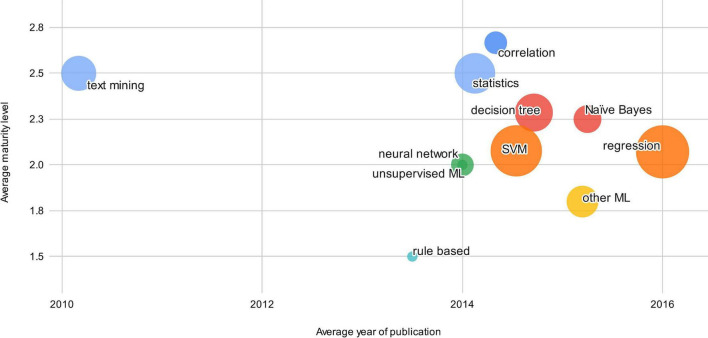
Decision technology concepts.

The majority (*n* = 15) of the investigated research uses a neural network approach (including support vector machines) in their systems. In 11 cases, support vector machines (SVM) were used. Other algorithms used were regressions, decision trees, random forest and rule-based approaches. We observed that 20 research papers did not have or mention any user interaction but worked solely on secondary data. The others used questionnaires or surveys, virtual humans or virtual reality. McWorther et al. proposed using temperature control, aromatherapy and auditory therapy capabilities for user interaction (36). Concerning maturity levels, AI algorithms are still mostly on maturity level two. Most advanced in terms of maturity were statistical methods and text mining methods, as indicated in Figure 7. The categories “statistics” and “machine learning” (ML) arose because some studies mentioned only these broad categories without further specifics.

### Data synthesis validation (EQ3)

The majority (*n* = 23) of articles validated the accuracy of the DDSS studied. Three articles validated user acceptance, two validated efficacy and three did not mention validation. Comparing algorithmic validation among research papers was difficult since a variety of scores, such as F1 scores, area under the receiver operating curve (63) or overall accuracy, were used and they cannot be converted. To be able to provide an estimation of how well current DDSSs perform, we extracted all accuracy measurements present in each paper and aggregated each scale individually. The mean accuracy (*n* = 11) of the DDSSs is μ = 82.2% with a median of η = 82% and a standard deviation of σ = 0.095. The mean area under the curve value (*n* = 8) is μ = 0.845 with a median of η = 0.84 and a standard deviation of σ = 0.064.

### Data synthesis user groups (EQ4)

The user groups mentioned were patients, clinicians and supporters of patients; however, the majority of papers did not explicitly mention specific user groups for their systems. Research covering decision support systems with higher maturity levels (four and above) included this information. Research dealing with decision support systems with lower maturity often lacked a clear user group since the process of using the proposed systems was not defined at that stage.

### Data synthesis medical domain (EQ5)

In addition to PTSD, which was tackled by all 30 research papers, four investigated depression (46–48, 55), two anxiety (34, 48) and one paranoia (58).

### Data synthesis decisions supported (EQ6)

Research focusing on predicting PTSD or its symptoms was most common (*n* = 11). Six papers focused on screening (35, 38, 45, 46, 50, 55) and six on treatment (32, 36, 43, 51, 53, 56). Four papers investigated the diagnosis of PTSD (37, 41, 52, 60) and five focused on monitoring PTSD (33, 35, 56, 58, 59).

### Data synthesis maturity level (EQ7)

**FIGURE 8:**
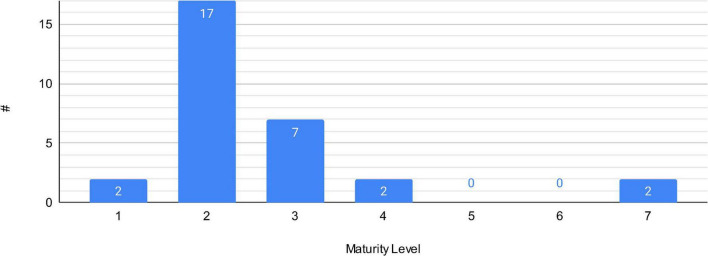
Bar chart – maturity levels.

The decision support systems were ranked according to the maturity scale described in see section “a framework for digital decision support systems.” As stated by answering research question two, the majority of papers work with secondary data. This is supported by the high volume of research with a maturity level of two. Figure 8 shows the number of articles grouped by maturity level.

## Discussion

This research highlights the state of the art in digital decision support systems for PTSD based on our proposed framework. We developed the framework to ensure a holistic overview of all features of a DDSS. The dimensions of the framework represent the topics of interest and the choice of features is based on the conceptualization of the terminology extracted from the included articles dimension by dimension.

Concerning the data dimension, we noticed that questionnaires and checklists are still the most common and most mature (see Figure 6) input for decision support systems. When examining clinical guidelines like NICE (64) for diagnosing PTSD, questionnaires and checklists are still the only approach mentioned for diagnostics. Even though some new technologies, such as virtual or augmented reality, were investigated in the research found in this review, we noticed an absence of input parameters based on smartphones or wearables like GPS sensors or accelerometers. We hypothesize that this is due to the short life cycle of modern technologies, making it difficult to offer clinical evidence of their benefits. Questionnaires and checklists, however, have been around for many years and the methodology for administering them has not changed, therefore there is more scientific evidence of their use. Researchers and medical professionals are more likely to research, invest and adopt technology with strong evidence. This could be another reason why DDSSs using new technology are not widely included in clinical processes.

The data dimension also showed that the sample size is on average small and the statistical significance of the results was not proven by the majority of the research articles. Several reasons contribute to this. In general, medical data are hard to obtain for research because secondary use is still not easy with many digital healthcare records and/or applications. Even if data can be obtained, they need to include the right parameters and have a structure that is usable for AI algorithms. Unstructured and text-based information is especially challenging to use for an AI. Further, most available datasets like the Jerusalem Trauma Outreach and Prevention Study do not include data on modern sensors (65).

The most common AI algorithm found during this literature review was support vector machines. Over the last few years, they have been developed to a *de facto* standard because they are easy to use, have good library support for programming and have low assumptions on the training data. We also observed that the number of research items resulting in usable products (maturity level ≥ 4) was low in three articles. Clinical studies with patient intervention (maturity level ≥ 3) were relatively low in nine papers out of 30. One reason for this could be that the small sample size of the research items does not provide sufficient evidence for clinical use.

All articles with a maturity level of 4 or more had, as one focus, validation of user acceptance and clearly defined user groups. Most articles with lower maturity levels did not have defined user groups. This could indicate a lack of strategic development and difficulties in bringing the research to a clinical setting. Our hypothesis is that interaction with users or integration into clinical processes is often much more challenging to solve than intelligence of cognition. Still, most papers focus on cognition, not user interaction; our framework’s validation dimension is evidence of this. We found 23 papers evaluating accuracy, which is an evaluation of AI technology, and five papers evaluating user acceptance or efficacy, meaning that they attempted to improve the current clinical process. Since most papers in our review are of maturity levels 1, 2 or 3 (meaning algorithm research), they do not include the clinical component necessary for user acceptance and efficacy evaluation. This shows a research gap when it comes to the enrichment of clinical processes with IT. The same goes for evaluating legal and IT-security constraints, which were not mentioned by any paper in our review. Since eHealth systems are getting increasingly focused by cyber attacks (66), IT and data security need to be a vital part of the evaluation to allow a safe DDSS adoption.

Further research has to be conducted on how the clinical process needs to be adapted for DDSSs to work, also in the context of the supported decisions. Most DDSS designers do not really understand the medical decision process but provide decisions in an “IT way.” One limitation of this general hypothesis is that our research focuses solely on DDSS for PTSD. However, the narrow approach to include only PTSD shows that even in a very well-scoped area, a DDSS is hard to implement.

Since we used an inductive research approach to design our framework based on currently available literature, some important framework dimensions might be missing. One example is that the framework includes many technical aspects of the implementations and fewer organizational and financial perspectives. We encourage further research to include dimensions that describe the adoption of DDSSs in clinical processes.

Introducing our novel framework for DDSS, we provide a guide for decision support system evaluation. The framework is complementary to other healthcare technology evaluation methods (clinical, organizational, financial) and thus supports the design of comprehensive evaluation systems for DDSSs. Applying the maturity dimension helped us to examine what features of a DDSS are present, thereby indicating the steps to take in order to move up in maturity when developing decision support systems. Since the framework was developed out of general considerations, it can be applied to decision support systems outside of PTSD or mental health. However, it should be further evaluated to examine whether the terminology suits other domains. Higher maturity scales in particular need additional verification, since only two papers in our review had a maturity level above 4.

## Conclusion

Our research aimed to analyze existing decision support systems for PTSD. Based on this goal, we developed a generic framework covering all dimensions of digital decision support systems. Our framework not only accelerates the development and benchmarking of DDSSs, but also acts as the foundation for our systematic literature review. Extracting data for all framework dimensions ensures consistency in our analysis and gives a holistic overview of DDSSs. During our review, we found working DDSS prototypes for PTSD and described their components. However, most of the systems are not evaluated in production use; they are only algorithmic models based on secondary datasets. This shows that there is still a gap between technical possibilities and actual clinical work. We proposed some possible explanations: small sample size, missing domain expertise, lack of focus to bring research to production. However, this gap should be analyzed further by testing our hypothesis and examining it with data from research on DDSSs for other mental diseases. For now, we conclude that only a few rare DDSSs for PTSD are ready for large-scale adoption in healthcare. The long-promised revolution of AI and ML for diagnosis in psychiatry, at least for PTSD, is yet to come.

## Data availability statement

The original contributions presented in this study are included in the article/supplementary material, further inquiries can be directed to the corresponding author.

## Author contributions

MB: conceptualization, methodology, investigation, resources, data curation, and writing – original draft. JM: investigation, data curation, and writing – original draft. PR: writing, review, editing, and supervision. All authors contributed to the article and approved the submitted version.
